# Functional dissection of the gene regulatory mechanism underlying variation in blood CD34^+^ cell levels at 1p36.23/*ENO1*

**DOI:** 10.1038/s41598-025-30068-4

**Published:** 2025-11-29

**Authors:** Zain Ali, Caterina Cafaro, Ludvig Ekdahl, Antton Lamarca, Aitzkoa Lopez de Lapuente Portilla, Björn Nilsson

**Affiliations:** 1 Division of Hematology and Transfusion Medicine, Department of Laboratory Medicine, BMC B13, Lund University, Lund, SE-221 84 Sweden; 2 Lund Stem Cell Center, Lund University, Lund, SE-221 84 Sweden; 3 Division of Hematology, Helsingborg Hospital, Charlotte Yhléns gata 10, 251 87 Helsingborg, Sweden; 4 Broad Institute, 415 Main Street, Cambridge, MA 02142 USA

**Keywords:** Hematopoietic stem and progenitor cells, Stem cell transplantation, Gata-2, Genetics, Gene regulation, Population genetics, Quantitative trait

## Abstract

Understanding the genetic regulation of blood CD34^+^ cell levels could lead to better methods for stem cell mobilization and transplantation. Recently, in a genome-wide association study (GWAS), we identified 11 genetic associations with blood CD34^+^ cell levels. One of the most significant maps to an intergenic region between the *ENO1* and *RERE* genes at chromosome 1p36.23. However, the underlying mechanism remains unknown. Here, using functional fine-mapping, we identify rs10864368:C > T as a causal non-coding variant. Specifically, the rs10864368-T allele abrogates a binding site for the GATA2 transcription factor, leading to downregulation of *ENO1* and *RERE* and lower CD34^+^ cell levels. Our data link a specific GATA2 target to the regulation of CD34^+^ cell levels, expanding on the role of GATA2 in human hematopoieisis.

**Supplementary Information:**

The online version contains supplementary material available at 10.1038/s41598-025-30068-4.

## Introduction

Circulating CD34^+^ hematopoietic stem and progenitor cells (HPSCs) are routinely used for stem cell transplantation^1^. These cells are harvested through leukapheresis of peripheral blood. Understanding the genetic regulation of blood CD34^+^ cell levels could expose new drug targets for stem cell mobilization and new insight into stem cell biology in humans. As baseline levels of CD34^+^ cells in peripheral blood are an important predictor of stem cell mobilization, better genetic understanding of these cell populations could be clinically relevant^2^.

**Fig. 1:**
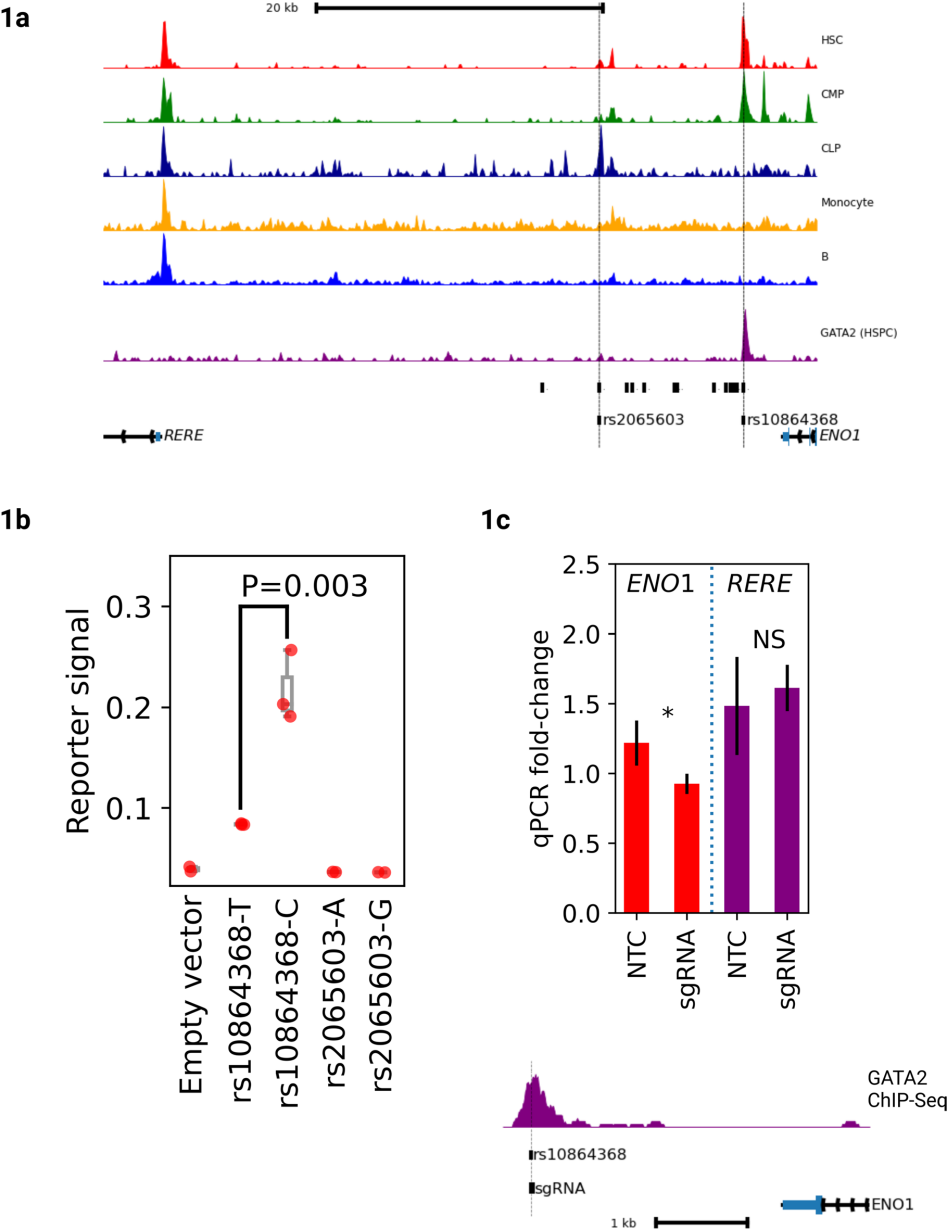
rs10864368 is the causal variant for the 1p36.23 association (**a**) *Top*: ATAC-Sequencing data^7^ from 5 different primary cell types and ChIP-Sequencing data^6^ for GATA2 in primary HSPCs. HSC: Hematopoietic stem cell, CMP: common myeloid progenitor, CLP: common lymphoid progenitor. Y-axis represents normalized read depth averaged over 500 bp windows. *Middle*: Unlabelled black bars represent 17 high LD variants corresponding to the 1p36.23 association. Dotted vertical lines show 2 variants directly intersecting chromatin accessibility peaks in primary HSPCs. *Bottom*: Gene diagrams showing locations of *ENO1* and *RERE*. (**b**) Luciferase reporter data comparing alleles of rs2065603 and rs10864368 in K-562 cells (3 biological replicates). Y-axis shows mean luciferase/renilla signal ratios and error bars show standard deviation across biological replicates. rs10864368-C shows significantly higher reporter signal than rs10864368-T (two-sided *t-*test *P* = 0.003). Neither allele of rs2065603 shows any expression above background. (**c**) CRISPR-Cas9 deletion of rs10864368 in K-562 cells (3 biological replicates). *Top*: qPCR data comparing sgRNA deleted cells to non-targeting controls (NTCs) for *ENO1* and *RERE*. Y-axis shows mean qPCR fold-change across biological replicates and error bars show standard deviation. Statistics shown are for one-sided *t-*test: *P ≤ 0.05, NS not significant. *Bottom*: Genome plot showing location of sgRNA relative to rs10864368 and GATA2 binding peak.

In a genome-wide association study encompassing 13,167 individuals from Sweden, we recently identified 11 independent genetic associations with blood CD34^+^ cell levels^3^. One of the most significant associations mapped to an intergenic region between the genes *ENO1* (Enolase-1) and *RERE* (Arginine-Glutamic Acid Dipeptide Repeats) on chromosome 1p36.23 (lead variant rs2047094; minor allele frequency = 0.49, *P* = 1.3 × 10^− 15^, β = −0.102 with blood CD34^+^ cell levels). *ENO1* catalyzes the conversion of 2-phosphoglycerate to phosphoenolpyruvate, the penultimate step in glycolysis. *RERE* encodes a transcriptional coactivator for the retinoic acid receptor^4,5^. Through expression quantitative locus (eQTL) analysis, we found that the rs2047094-A allele (which confers higher blood CD34^+^ cell levels) is associated with higher expression of *ENO1* and, to a lesser extent, *RERE* in primary blood CD34^+^ cells. Consistent with this, promoter capture Hi-C data for blood CD34^+^ cells^6^ revealed chromatin looping interactions with the *ENO1* and *RERE* promoters. Our previous work thus indicates that the genetic association at 1p36.23 with CD34^+^ cell levels is driven by cell-autonomous changes in *ENO1* and/or *RERE* expression in HSPCs. However, the 1p36.23 association is represented by 17 variants in high linkage disequilibrium (LD) spanning a ~ 14 kB region (Fig. 1a) and the variant underlying the changes in gene expression remains unknown. To address this, we conducted a functional fine-mapping study aimed at pinpointing the causal variants and their mechanisms of action.

## Methods

### Cell culture

All assays were performed using K-562 erythroleukemia cells (DSMZ, #ACC 10) which are routinely used to study HSPC function and are naturally heterozygous for rs10864368. K-562 cells were cultured in RPMI 1640 supplemented with 10% heat-inactivated FCS (Gibco) at 37 °C with 5% CO2 and maintained at a density of 0.5–1.0.5.0 million cells/mL.

### ATAC-Seq and ChIP-Seq data analysis

Chromatin accessibility data for primary blood cell types was accessed using GEO (GSE74912)^7^. GATA2 and SMAD1 ChIP-Seq data for primary HSPCs were accessed using GEO (GSE60793, GSE104574)^8,9^. Chromatin accessibility and ChIP-Sequencing data for K-562 cells were accessed using ENCODE for allelic imbalance analysis^10^. Raw FASTQ files were aligned to the hg38 human genome assembly using HISAT2. BAM files were analyzed using IGV to identify heterozygous samples and extract read counts at rs10864368. In cases of allelic imbalance, counts of both alleles were used to evaluate the statistical significance of imbalance using a binomial test.

### Luciferase assay

We performed luciferase assays for two candidate variants (rs2065603 and rs10864368). For rs2065603, we constructed 900-bp DNA sequences centered on the variant (Integrated DNA Technologies). For rs10864368, due to synthesis problems, we instead adopted a PCR strategy and amplified an 864-bp sequence containing rs10864368 from genomic DNA extracted from K-562 cells, which are naturally heterozygous for this variant (see Supplementary Table 1 for full reporter sequences). We screened PCR clones using Sanger sequencing until both alleles were recovered. Both sequences were subsequently cloned into the pGL3-Basic vector (E1751, Promega). Reporter plasmids were electroporated into K-562 cells using the Neon electroporation system (Thermo FisherScientific). Luciferase and renilla activities were measured 24 h after electroporation using DualGlo Luciferase (Promega, #E1960) on a GLOMAX 20/20 Luminometer (Promega).

### CRISPR/Cas9 deletion of variant region

To delete the rs10864368-harboring region, we used a single guide RNA (sgRNA) directly spanning the variant (Supplementary Table 2). The sgRNA was cloned into SpCas9(BB)−2 A-GFP PX458 vector (Addgene, #48138) and electroporated into K-562 cells using the Neon electroporation system (Thermo FisherScientific). GFP-positive cells were sorted 24 h after transfection. RNA was extracted from the same cells, reverse-transcribed, and quantified by Taqman assays (Hs00201558_m1 for *RERE*; Hs00361415_m1 for ENO1; Hs01060665_g1 for ACTB) using the StepOnePlus qPCR instrument (Applied Biosystems).

### Motif analysis

For motif analysis we used PERFECTOS-APE^11^ to scan the HOCOMOCO database of transcription factor binding motifs to predict what TF motifs would be most likely to be disrupted by rs10864368. As a complementary approach, we calculated binding efficiencies following a recently described method^12^. We obtained the GATA2 position weight matrix (PWM) from JASPAR database (MA0036.3). For each allele, we extracted a 51 bp genomic sequence centered on the variant position (hg38 chr1:8858254) and scanned all possible 11 bp windows overlapping the variant on both forward and reverse strands. The PWM score for each sequence was calculated as the sum of position-specific log-odds ratios: Score(s) = sum from i = 1 to n of log2(M_si, i/b_si), where s is the DNA sequence of length n (11 bp for GATA2), M_si, i is the frequency of nucleotide si at position i in the PWM, and b_si is the background frequency (0.25 for uniform nucleotide distribution). Binding efficiency was calculated as 2^(PWM score), representing the relative binding affinity. The fold change in binding efficiency between alternate and reference alleles was computed as the ratio of their binding efficiencies. The sequence with the highest PWM score overlapping the variant position on either strand was selected as the best match for each allele.

## Results

Our initial GWAS findings identified a LD block of 17 linked variants but could not determine which of these is directly causal for altered HSPC levels. As non-coding variants often act by altering transcription factor binding, we prioritized candidate causal variants that intersect with accessible chromatin in HSPCs. Using ATAC-sequencing data for 18 cell types derived from blood and bone marrow^7^, we found that two of the 17 associated variants (rs10864368 and rs2065603; *r*^2^ = 0.996 and 0.9034 with rs2047094, respectively) map to accessible chromatin of primary CD34^+^ cell types, including hematopoietic stem cells (HSC), common myeloid progenitors (CMP), and common lymphoid progenitors (CLP) (Fig. 1a). Particularly, the rs10864368-harboring region is highly accessible in HSCs. To assess the transcriptional activity of the two candidate variants, we performed luciferase reporter assays in K-562 leukemia cells. Luciferase constructs harboring rs10864368-C, which confers higher *ENO1* and *RERE* expression and higher blood CD34^+^ cell levels, showed higher activity than rs10864368-T constructs (two-sided Student’s *t*-test, *P* = 0.003).

**Fig. 2:**
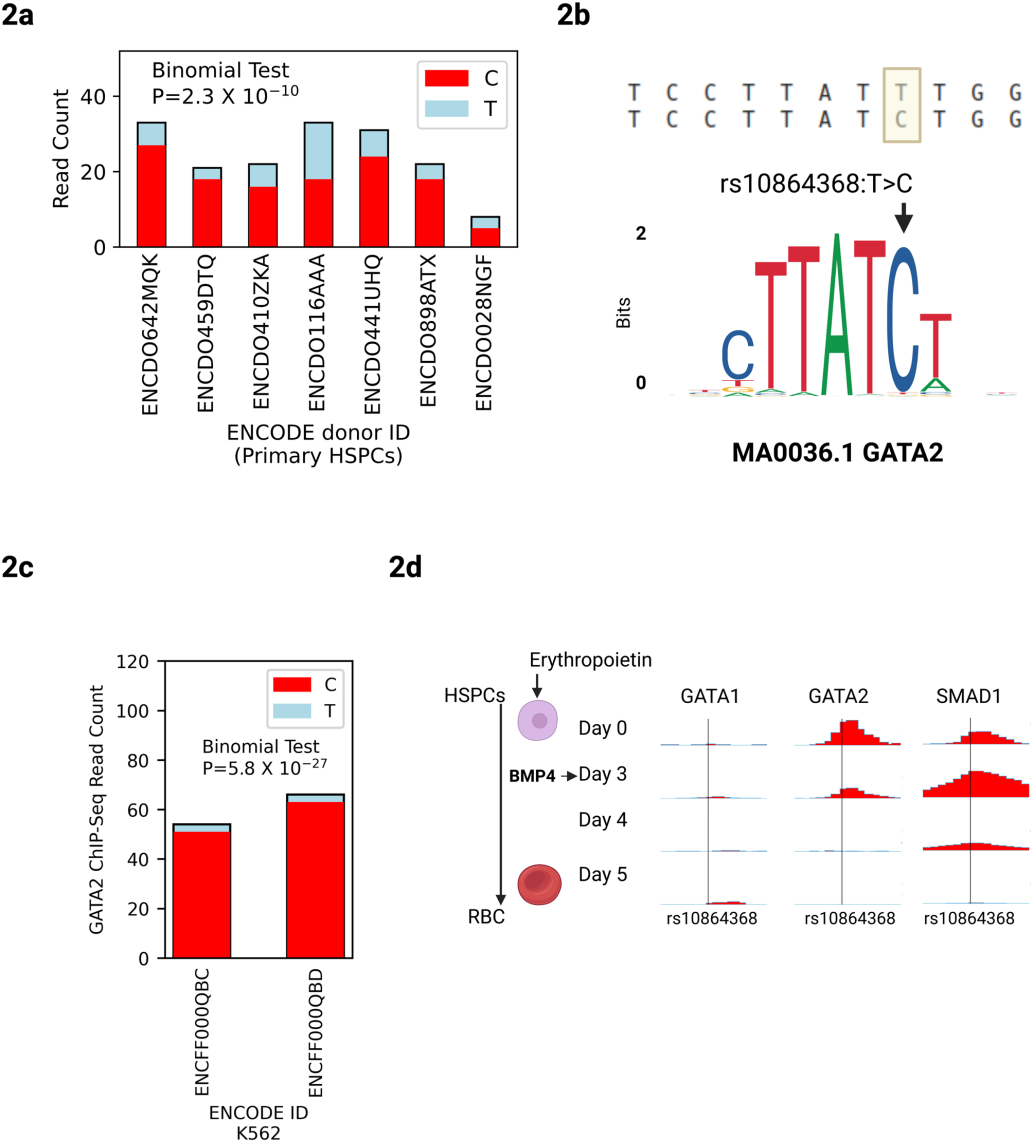
rs10864368-T reduces chromatin accessibility and GATA2 binding. (**a**) ENCODE DNase-Sequencing data in primary CD34^+^ HSPCS from 7 heterozygous donors^7^, showing allelic read counts for rs10864368 as stacked bar plots, showing significant allelic imbalance towards rs10864368-C across all donors. (**b**) *Top*: Local sequence context around rs10864368 highlighting the location of the variant. *Bottom*: JASPAR binding motif for GATA2 showing position affected by rs10864368. (**c**) ChIP-Sequencing data for GATA2 in K-562 cells, showing read counts for rs10864368 as stacked bar plots, for 2 technical replicates. K-562 cells are naturally heterozygous for rs10864368 and show significant imbalance towards rs10864368-C. (**d**) Time-course ChIP-Sequencing data showing temporal dynamics of GATA1, GATA2 and SMAD1 binding at rs10864368 during ex vivo differentiation of primary HSPCs to red blood cells. Y-axes show normalized read depth averaged across 50 bp windows.

At the same time, we could not see a difference between rs2065603-A and rs2065603-G constructs (Fig. 1b). To obtain further support for a regulatory relationship, we disrupted the rs10864368-harboring region in K-562 cells by use of CRISPR/Cas9 with a single guide RNA (sgRNA) directly overlapping the variant. We previously observed a positive correlation between chromatin accessibility at rs10864368 and *ENO1* expression and so tested the hypothesis that this regulatory element acts as a transcriptional activator. Confirming our hypothesis, CRISPR deletion resulted in lowered *ENO1* expression (one-sided Student’s *t*-test, *P* = 0.042) but, consistent with our previous study showing a weaker eQTL on *RERE* than for *ENO1*, we observed no significant change in *RERE* expression (Fig. 1c). Thus, our data primarily point to ENO1 being the main candidate gene, though we cannot rule out a role for *RERE* without further functional work. To characterize the effects in an endogenous chromosomal context, we examined allelic imbalance in chromatin accessibility at rs10864368 in ENCODE DNase-Seq data from primary blood CD34^+^ cells from seven heterozygous donors, observing a significant bias towards rs10864368-C (Fig. 2a; two-sided binomial test *P* = 2.3 × 10^− 10^). Altogether, these data identify rs10864368 as a likely causal variant, with rs10864368-C associating with increased *ENO1* expression, local chromatin accessibility and blood CD34^+^ cell levels.

Next, we searched for transcription factors mediating the effects of rs10864386 on *ENO1* expression. Using motif analysis, we found that the most significantly perturbed motif was for GATA2 (Fig. 2b), with rs10864368-T disrupting a canonical GATA motif (*P*-value fold-change: 291.01)^11^. To further validate this prediction, we used a complementary approach^12^ to calculate binding affinities for both alleles based on the GATA2 position weight matrix (JASPAR MA0036.3) and find that the C allele demonstrated a 2,012-fold increase in binding efficiency compared to the T allele. GATA2 has an extensively characterized role in stem and progenitor cell biology. In mice, conditional deletion of *Gata2* in hematopoietic cells leads to complete loss of adult HSPCs^13^. In humans, germline mutations in *GATA2* lead to GATA2 Deficiency, a heritable disease that combines immunodeficiency with myelodysplastic syndrome, and somatic mutations are observed in 1–4% of patients with myeloid malignancies^14,15^. ChIP-Seq data^8^ show GATA2 binding directly to rs10864368 in primary CD34^+^ HSPCs (Fig. 1a).

To investigate the effect of rs10864368 on GATA2 binding in an endogenous genomic context, we took advantage of the fact that the K-562 cell line is naturally heterozygous for this variant. We examined allelic imbalance for GATA2 binding at this site using public ENCODE ChIP-Seq data^10^. We observed a significant bias towards rs10864368-C (Fig. 2c, percent C reads: 95%, H_0_: *p* = 0.5, two-sided binomial test *P* = 5.8 × 10^− 27^), further supporting that rs10864368-T disrupts GATA2 binding in the native genomic context. Although K-562 cells are triploid at many loci, whole-genome sequencing data from ENCODE indicate that the region containing rs10864368 is tetraploid. Furthermore, the degree of imbalance is so severe that the binomial test would still be significant in either triploid heterozygous genotype (H_0_: *p* = 0.33, two-sided binomial test *P* = 1.3 × 10^− 13^; H_0_: *p* = 0.66, two-sided binomial test *P* = 1.2 × 10^− 45^). We conclude that rs10864368-T abrogates a GATA2 binding site and reduces local chromatin accessibility in primary HSPCs.

We investigated the developmental dynamics of GATA2 binding at rs10864368 in time-course ChIP-Seq data from primary HSPCs differentiating to red blood cells^9^. GATA2 disappears from the site over the course of erythroid differentiation, but is not displaced by GATA1 as the cell commits to an erythroid fate (Fig. 2d**)**. The signalling TF SMAD1 also transiently binds this site, indicating that the regulatory element integrates BMP signalling with lineage-determining factors like GATA2. Regulatory elements of this kind that integrate signalling and lineage-determining TFs have been shown to be enriched for GWAS variants^9^.

Finally, we searched for all other significant phenotypes associated with rs10864368 in GWAS data^16^ and find that rs10864368-C is associated with increased frequency of myeloid lineage cells such as neutrophils, and with reduced lymphocyte percentage (Table 1)^17–20^. This suggests that increased GATA2 binding at rs10864368 leads to expansion of myeloid lineage HSPCs such as CMPs, leading to an increase in circulating CD34^+^ cells and biasing hematopoiesis towards the myeloid lineage.

**Table 1:** Known GWAS trait associations with rs10864368

**Trait**	**P**	**Beta**	**PMID**
Plateletcrit	6.59-58	0.0337624	32888494
Platelet count	6.45E-36	0.022872	32888493
Neutrophil count	4.01-35	0.023683	32888493
White blood cell count	1.15E-31	0.021532	32888493
Neutrophil percentage of white cells	7.8E-14	0.0160352	32888494
High light scatter reticulocyte count	8.1E-13	0.0131213	34226706
Lymphocyte percentage of white cells	1E-11	-0.0145583	32888494
Granulocyte count	6.8E-11	0.0233531	27863252
Intrinsic epigenetic age acceleration	1.09E-10	-0.201	34187551
Sum basophil neutrophil counts	1.16E-10	0.0230463	27863252
Sum neutrophil eosinophil counts	1.23E-10	0.0230015	27863252
Myeloid white cell count	3.78E-10	0.0224726	0.0224726
Basophil count	5.59E-10	0.012657	32888493

## Discussion

Understanding the genetic regulation of blood CD34^+^ cell levels could have potential clinical implications for stem cell harvesting and transplantation and provides insights into HSPC physiology in humans. Our data support that the 1p36.23 association with blood CD34^+^ cell levels is the result of rs10864368-T decreasing GATA2 binding and local chromatin accessibility, leading to downregulation of *ENO1* and *RERE* in primary human CD34^+^ cells, though the downstream mechanisms of these transcriptional changes remain to be elucidated.

We note that our work has technical limitations that caution against the conclusion that rs10864368 is the only causal variant for this association. Cancer cell lines such as K-562 are imperfect surrogates for primary HSPCs and future work should evaluate GWAS variants in primary cells. Additionally, the CRISPR editing strategy we employed is suboptimal as it employs a single cut site without directly assessing editing efficiency through sequencing. While our experiments provide limited in vivo evidence linking the regulatory element to *ENO1* expression, precise editing through homology directed repair or base editing would provide stronger evidentiary support for the functional impact of rs10864368 in its native genomic context. As many eQTL associations are driven by multiple causal variants, without more comprehensive genome editing experiments in primary HSPCs we cannot completely rule out that other linked variants are responsible for downstream changes in gene expression and HSPC frequency.

Intriguingly, our PheWAS analysis revealed that rs10864368-C is also associated with reduced intrinsic epigenetic age acceleration (Table 1), a measure of epigenetic aging derived from DNA methylation data. As HSPC function has been directly linked to epigenetic age acceleration, the mechanistic link between altered *ENO1* or *RERE* expression and stem cell aging could be an interesting avenue for future research^21,22^.

While our gene expression data and CRISPR-Cas9 results primarily point to a role for *ENO1*, both *ENO1* and *RERE* remain plausible candidate genes based on eQTL data. As a glycolytic enzyme, *ENO1* expression could influence the balance between glycolysis and mitochondrial respiration. The regulation of energy metabolism is known to play a key role in HSPC function^23^. *RERE* is a co-regulator of retinoic acid signaling^4,5^ and animal models have shown that reduced retinoic signalling leads to reduced HSPC numbers^24^.

Of note, GATA2 is a central regulator of hematopoietic stem and progenitor cell function and a causal factor in several blood disorders. Here, we link a specific GATA2 target variant to the regulation of blood CD34^+^ cell levels, which has previously not been reported. In conclusion, our work expands on the role of GATA2 in human hematopoiesis.

## Supplementary Information

Below is the link to the electronic supplementary material.

Supplementary Material 1

Supplementary Material 2

## Data Availability

Data is provided within the manuscript or supplementary information files. All data are contained within the paper.
